# Translation, cultural adaptation and psychometric testing of short form quality of trauma patient experience measure (SF-QTAC-PREM) to a Swedish context

**DOI:** 10.1371/journal.pone.0342088

**Published:** 2026-03-16

**Authors:** Elizabeth Mårtenson, Anna Kullberg, Anders Enocson, Katarina E Göransson

**Affiliations:** 1 Department of Medicine Solna, Karolinska Institutet, Stockholm, Sweden; 2 Department of Trauma, Acute Surgery and Orthopaedics, Karolinska University Hospital, Stockholm, Sweden; 3 Department of Oncology-Pathology, Karolinska Institutet, Stockholm, Sweden; 4 Department of Molecular Medicine and Surgery, Karolinska Institutet, Stockholm, Sweden; 5 School of Health and Welfare Department of Caring Sciences, Dalarna University, Falun, Sweden; 6 Emergency and Reparative Medicine Theme, Karolinska University Hospital, Stockholm, Sweden; University of Calgary Cumming School of Medicine, CANADA

## Abstract

Patients admitted to hospital due to trauma present with a multifaceted constellation of injuries, severity of injuries and comorbidities that require a complex and specialised somatic and psychological care system. Good care processes have been linked to better recovery and improved long-term health and health-related quality of life. There is both a knowledge gap around quality and safety of patient’s care following injury, as well as a lack of a validated questionnaire to systematically collect trauma patients’ experiences. The aim of this study was to translate, culturally adapt and determine the psychometric properties of the SF-QTAC-PREM acute care for use in a Swedish population of trauma patients. A prospective, cross-sectional study was performed in which patients at a Level 1 trauma centre in Sweden participated. Translation and adaptation was performed in accordance with the ISPOR and WHO guidelines, which generated a 22-item questionnaire. Thirty patients completed the test-retest, and a further 150 patients the psychometric testing. Reliability was established using Intraclass Correlation Coefficient (ICC), and weighted Cohen’s Kappa coefficient, which showed a moderate to substantial agreement, and good reliability. The psychometric properties of the questionnaire using Exploratory Factor Analysis identified a two-factor solution with two stand-alone items. A good internal consistency was shown between factors using Cronbach’s alpha (0.85 Information and care, 0.84 Patient safety), concluding that the factors were not able to be combined. Spearman’s R correlation showed weak (0.31) and moderate (0.58) positive relationships between the items and global rating scale contributing to a good overall construct validity. The new Swedish SF-QTAC-PREM acute care exhibited above acceptable external reliability, good overall internal consistency and construct validity.

## Introduction

Injury accounts for over four million deaths a year worldwide [1], with millions more people with non-fatal injuries presenting to emergency departments or other health care facilities annually [2]. In 2024, around 12 000 patients were registered in the national Swedish trauma register (SweTrau) after being admitted to hospital in conjunction with a primary trauma-team alert, or secondary alert with a NISS (New Injury Severity Score) >15 [3]. Most of these patients were men (65%), mainly in working age between 18–64 years (56%), with almost a third (30%) being over 64 years of age. Traffic accidents and falls were the most common mechanisms of injury [3].

Patients admitted to hospital due to trauma present with diverse injuries with varying severity and underlying comorbidities, which often require complex and specialised somatic and psychological care. Thus, high-quality trauma care needs to be individual, person-centred and support specific recovery needs [4]. After discharge from hospital, many trauma patients have continued care needs, often provided via primary healthcare, hospital outpatient units and/or with support from carers or relatives [3,5]. Previous studies have shown that many trauma patients are not adequately prepared for discharge regarding post injury pain control [6,7] and mobilization [8,9], and also lack information and guidance for wound care [10]. Swedish studies reported that discharge needs of patients were often not sufficiently met [10], contributing to poor outcomes related to pain control, mobility and mental health issues, including post-traumatic stress disorder (PTSD) [11].

Quality and safety of care can be improved by incorporating patient’s experience of care in evaluations of health care services [12–14]. Good care processes have been linked to better recovery and positive consequences for longer term health and health-related quality of life [15–19]. Positive correlations have been found between PREM (Patient Reported Experience Measures) results and patient outcomes [20]. PREM instruments are either general/non-specific and validated for a broad population (e.g., Picker Patient Experience – PPE 15 [21] or specific, e.g., for cancer [22,23]. Applying questionnaires suitable for a specific population have been shown to ensure reliability in specific target groups [24].

In Sweden, validated PREMs such as the PPE-15 [21,25] and the Swedish National Patient Survey (NPE) are widely used to capture general patient experiences across diverse clinical settings. However, these instruments are not trauma-specific and therefore lack sensitivity around acute care processes, recovery from surgical or orthopedic trauma surgery or elements which are more specifically important to improve trauma patients’ outcomes. This lack of specificity limits the ability to evaluate whether processes to increase safety and quality of care actually occur, and limits patient participation in contributing to improvements in care processes or services [25].

In Canada, the Quality of Trauma Care Patient Reported Experience Measure (QTAC-PREM), was developed in 2016 [26]. Since then, QTAC-PREM has been validated and modified into a short form for the acute phase (SF-QTAC-PREM acute care) [27]. This questionnaire has been used in Canada, the US and England [5,28]. The SF-QTAC-PREM acute care comprises 24 items grouped across three sections: “During your care for this injury” (14 items), “Overall care” (4 items) and “Questions about you” (6 items). The structure of the questionnaire varies, including numeric rating scale, Likert scales with responses of either 3, 4 or 5 response alternatives, categorical response alternatives, and free text comments [27].

Currently, there is no known systematic evaluation of trauma patients’ experience or needs at discharge in Sweden, nor a validated PREM instrument to systematically collect trauma patients’ experiences. Development of a validated trauma specific PREM in Swedish could fill this knowledge gap and thereby identifying local/hospital/regional and even national differences in care quality and identify patient safety and care needs in trauma inpatient care and during discharge processes.

The aim of the study was to translate, culturally adapt and determine the psychometric properties of the SF-QTAC-PREM acute care for use in a Swedish population of trauma patients.

## Materials and methods

This was a prospective, cross-sectional study that was carried out in three phases during 2023–2025.

### Setting and study participants

The study setting was a specialized trauma ward at a Level 1 trauma center at a university hospital in Sweden. The ward has 28 beds, 6 of which support high dependency care for newly injured or stepdown intensive care trauma patients. Five registered nurses and 5 nurse assistants provide care for the patients on each day/evening shift, in collaboration with a team of 5–6 specialist physicians, and therapists from other disciplines, e.g., physiotherapists, occupational therapists. The intensity of care at the ward is high, caring for severely injured patients with complex needs.

### Eligibility criteria

Patients who were eligible for participation were 18 years of age or over, primary admitted to the hospital after a trauma (according to the national trauma alert criteria), or via secondary transport from other hospitals. All kinds of adult trauma patients are admitted to this unit, including some patients with isolated complex orthopaedic injuries.

Additional inclusion criteria included: hospital length of stay over 36 hours, a Swedish social security number, enough proficiency in the Swedish language to understand instructions, ability to give informed consent to participate and to complete the written questionnaire. Patients who were excluded included patients having severe cognitive impairment, severe exacerbations of psychiatric illness, or patients who had attempted suicide or were injured due to self-harming behaviour.

The number of patients needed in the three phases were estimated by the WHO methods for face validity determination [29], test-retest [30] and psychometric analyses [31,32].

### Phase 1: Translation and cultural adaption

The translation and cultural adaptation of the questionnaire and was performed using two guidelines: The Cross-Cultural Adaptation Process for Patient-Reported Outcomes Measures by ISPOR [33] and the Guidelines for translation and cultural adaptation by the World Health Organization [29]. Forward translation was performed by researchers experienced in translation and validation studies, with focus on keeping the style close to the English language version [34]. An expert group consisting of bilingual (Swedish and English) trauma specialists from nursing and medical disciplines reviewed the translation for inconsistencies with linguistic content into the Swedish context, and in relation to a Swedish clinical health care provision context. An external translation specialist was commissioned to perform the back translation. Discrepancies were discussed and amended after further discussion. Following the back translation process, face validity of the SF-QTAC-PREM acute care was evaluated through individual semi structured cognitive interviews with ten patients, using think aloud methodology [35], with final minor linguistical adjustments concluding the process. When asked, patients did not think that there were any questions missing or that other items should be included in the measure.

### Phase 2: Test-retest

To evaluate the questionnaire’s reliability over time, 30 patients answered the questionnaire twice; once at discharge and once again within 7 days after discharge from hospital [36]. This provided time to reduce the possibility of recalling the previous test but short enough to avoid a true change in health status of trauma patients [37,38].

### Phase 3: Psychometric testing

In the third phase of the study, responses from 150 patients with complete data sets were used to assess the questionnaire’s psychometric properties.

### Data collection

Identification and recruitment of patients, according to the inclusion criteria, was carried out by one of the authors (EM) for phase 1,2 and 3. In phase 3, one additional staff member who had been informed about the research project also identified and recruited participants. In all phases, recruitment of patients occurred prior to discharge and after verbal informed consent was received upon participation. All patients in phases 2 and 3 were offered to answer the questionnaire either online or in paper form. Paper responses were handed to the researcher (EM) after completion of test questionnaire. Re-test questionnaires were provided with a postage paid envelope, addressed to the researcher at the hospital address. Data collection in phase 1 occurred between 6 June 2023 and 27 June 2023, and phase 2 and 3 data collection commenced on 7 August 2023 and was completed on 10 March 2025. Consent for participation in all three phases of the project was obtained verbally, and completion of the interview or questionnaire was considered as providing consent for participation in the project. According to the ethical approval and Swedish legal regulations, no witness was necessary for gaining this consent.

### Data analysis

Test-retest reliability was assessed for all items in the original two subscales and the two stand-alone items using Cohen’s weighted kappa (k) coefficient for items for dichotomous data and Intraclass Correlation Coefficient (ICC) (two-way random effects model) for ordinal scale data [39]. Cut-off values for Cohen’s weighted kappa coefficient were; 0 no agreement due to random chance, > 0–0.2 slight agreement, > 0.2–0.4 fair agreement, > 0.4–0.6 moderate agreement, > 0.6–0.8 substantial agreement, and >0.8–1.0 almost perfect agreement [40]. Cut-off values for ICC (based on a 95% confident interval) were; < 0.5 poor reliability, 0.5–0.75 moderate reliability, > 0.75–0.9 good reliability, and >0.9 excellent reliability [41].

*Psychometric testing* included all items in the two subscales and the two stand-alone items. The testing for a factor solution was performed by Exploratory Factor Analysis (EFA) while Cronbach’s alpha assessed internal consistency. Spearman’s rank correlation was used to examine the relationship between the items and the global rating scale to ascertain construct validity using cut of values of 0–0.9 no correlation, > 0.1–0.39 weak correlation, > 0.4–0.69: moderate correlation, > 0.70–0.89: strong correlation, > 0.90–1.0: very strong correlation [42]. Categorical data was presented as frequency and percent distribution. Numerical data was presented as median (range). The results were considered significant at p < 0.05. The statistical software used was IBM SPSS Statistics, version 25 for Windows (SPSS Inc., Chicago, Illinois) and Jamovi version 2.6.44 for windows (opensource jamovi desktop - jamovi).

Ethical approval was obtained from the Swedish Ethical Review Authority: Dnr: 2023-01933-01 and amendment approvals Dnr 2023-08019-02 and Dnr 2024-06553-02.

## Results

In total, 222 patients were invited to participate in the study, and finally 190 patients were recruited, yielding an overall response rate of 86%. In Table 1, the demographic information about participants in phase 2 and 3 is presented. The median (range) age for all patients was 59 (18–83) years and 144 (63%) were male. The most common injury mechanism in phase 2 (test retest) was traffic accident (27%, n = 8) followed by falls (21% n = 6) (Table 1). In phase 3 (test), this was reversed with more falls (45% n = 65) than traffic accidents (22% n = 15). Demographic data was not collected for patients in phase 1.

**Table 1:** Demographics of the study participants taking part in phase 2 and 3 (n = 180).

Phase	Phase 2 Test-retest n = 30	Phase 3 Test n = 150
**Age in years**, median (range)	57 (18-83)	54 (18-82)
	n (%)	
**Male gender**	15 (50)	99 (66)*
**Injury mechanism**	**	***
Car accident	5 (17)	12 (8)
Injured as a pedestrian	–	2 (1)
Fall	6 (21)	65 (45)
Cycle accident	5 (17)	13 (9)
Burn injury	–	–
Assault	1 (3)	7 (5)
**Other injury mechanism**	12 (41)	46 (32)
Ski accident	1 (8)	10 (31)
Motorcycle	3 (25)	9 (28)
Sporting or leisure	–	5 (16)
Other (no description)	–	5 (16)
Workplace/working at home Related to previous medical condition	4 (33) –	4 (13) 4 (13)
Shooting	–	3 (9)
Sharp objects at home	2 (17)	3 (9)
Explosion	–	1 (3)
Riding	2 (17)	1 (3)
Electric scooter	–	1 (3)
**Level of Education**	30 (100)****	150 (100)
No formal education	–	1 (1)
Secondary school education	4 (14)	23 (16)
High school	14(50)	52 (35)
University level education	10(36)	70 (48)

* 1 patient replied other, ** 1 missing data, *** 5 missing data, **** 2 missing data.

### Phase 1: Translation and cultural adaptation of the questionnaire

Minor changes were made after forward translation and the expert group review, including shortening question text (n = 2), and changes to specific terminology (n = 2). Two items deemed to be culturally irrelevant were removed from the demographic items (Ethnic orientation and Language spoken at home). During the back translation process, small linguistic issues were identified and amended. Linguistic changes were made in some questions (n = 5) and in some answer alternatives (n = 3). Conceptual equivalence was considered adequate, the original being simple language with terminology which was easy to understand. Face validity interviews ascertained that readability, and comprehensiveness was found to be acceptable. Consensus was found on most questions, with small amendments to linguistics (e.g., removal of the word *agitation*, which was regarded as unclear; addition of ‘patient’ to safety to specify the response and shortening two questions to improve clarity). Finally, the translation and cultural adaption process resulted in a 22-item Swedish questionnaire distributed across three sections: “During the care for the injury” (14 items),” Overall care” (4 items) and “Questions about the respondent” (4 items).

### Phase 2: Test-retest reliability

A total of 30 test-retest questionnaires were completed within seven days (two questionnaires per respondent). Table 2 illustrates the 15 items subject to data analyses. The Cohen’s weighted kappa coefficient showed moderate to substantial agreement. The ICC coefficients were moderate for three items (27%), good for six items (55%) and excellent for one item (9%) (Table 2). One item “Treated unfairly” had a poor relationship (n = 1, 9%).

**Table 2:** Test-retest reliability of SF-QTAC-PREM acute care (n = 30).

Item	Intraclass correlation coefficient – average measures (95% confidence interval)	Cohen’s weighted Kappa
1. All injuries explained		0.59 (0.32-0.86)
2. Explained self-care		0.64 (0.35-0.94)
3. Explained recovery time-line		0.65 (0.41-0.89)
4. Consistent information	0.60 (0.12-0.82)	
5. Pain well-controlled	0.65 (0.24-0.84)	
6. Helped with agitation	0.82 (0.61–0.92)	
7. Handled carefully	0.89 (0.77–0.95)	
8. Helped with hygiene	0.97 (0.93–0.99)	
9. Providers explained their roles	0.83 (0.64–0.92)	
10. Addressed concerns	0.72 (0.40-0.87)	
11. Dignity considered	0.88 (0.73–0.94)	
12. Offered to discuss emotional needs		0.74 (0.53-0.95)
13. Perceived safety of care	0.83 (0.63–0.92)	
14. Treated unfairly	0.29 (−0.53–0.66)	
15. Global rating	0.90 (0.77–0.96)	

### Phase 3: Psychometric testing

Exploratory factor analysis using a Promax rotation suggested two latent constructs with a two-factor model (13 items) and two stand-alone questions (“Offered to discuss emotional needs” and “Global rating”). Most (7/11) items were in Factor 1: *Information and care* demonstrated loadings >0.60 and none <0.51. Factor 2 *Patient safety*, which comprises the items “Perceived safety of care” and “Treated unfairly“ loaded at least 0.815. No cross loadings over 0,32 were indicated between factors. The collected ordinal data was unimodal, with five items having normal distribution and ten illustrating more skewed results. ICC and Kappa results with wider confidence intervals and lower score included the five items with normal distributions, and three items with skewed distributions. All items had moderate or higher agreement.

Internal consistency of factors demonstrated Cronbach’s alpha values of 0.85 (information and care) and 0.84 (patient safety) supporting the two-factor model [43] see Table 3.

**Table 3:** Psychometric measurement properties of the SF- QTAC-PREM acute care (n = 150).

Subscale Item	Sub-scale factor loadings	Sub-scale Cronbach’s alpha	Item to global rating item correlations Spearmans r (p-value)
**Factor Information and care**		**0.85**	
1. All injuries explained	0.61	0.84	0.59 (<.001)
2. Explained self-care	0.61	0.84	0.56 (<.001)
3. Explained recovery time-line	0.53	0.85	0.44 (<.001)
4. Consistent information	0.68	0.84	0.48 (<.001)
5. Pain well-controlled	0.57	0.84	0.41 (<.001)
6. Helped with agitation	0.62	0.84	0.47 (<.001)
7. Handled carefully	0.60	0.84	0.42 (<.001)
8. Helped with hygiene	0.55	0.85	0.38 (<.001)
9. Providers explained their roles	0.63	0.84	0.50 (<.001)
10. Addressed concerns	0.70	0.83	0.58 (<.001)
11. Dignity considered	0.52	0.84	0.49 (<.001)
**Factor Patient safety**		**0.84**	
13. Perceived safety of care	0.82	0.83	0.41 (<.001)
14. Treated unfairly	0.93	0.64	0.31 (<.001)
**Stand-alone item**			
12. Offered to discuss emotional needs	–	–	0.18 (0.028)
15. Global rating	–	–	–

The corelation analysis to examine the relationship between the items and the global rating scores and construct validity (Spearman’s r correlation) showed two items “helped with hygiene” and “Treated unfairly” having weak correlation and a third item “Offered to discuss emotional needs” having very weak correlation. The remaining 9 items displayed moderate correlation. All correlations but one were significant: the item “Offered to discuss emotional needs” demonstrated weak correlation (0.18, p = .028). This result supports the premise that the item should not be included in a factor but stands alone.

## Discussion

The aim of the study was to translate, adapt and determine the psychometric properties of the SF-QTAC- PREM acute care for use in a Swedish population of trauma patients. The new Swedish SF-QTAC-PREM acute care displayed good reliability, a two-factor structure, internal consistency and construct validity. In summary, the analyses showed this to be a valid measure of trauma patients’ experiences in this Swedish patient cohort.

During the cultural adaptation, a universalist approach to conceptual equivalence was used in the translation – i.e., culture will have some effect on the construct being measured, but the construct should be comparable [44]. No new constructs were identified in the translation process and only minor changes were made. The expert group explored multiple meanings, grammatical errors, use of idioms and semantic equivalence in the translation, which can be problematic to translate. Cultural adaptation included trying to capture the experience of going through trauma care in Sweden and no items were removed from the sections around the patients’ experience of care, i.e., “During your care for this injury” or “Overall care”. However, two items from the “Questions about the respondent” section was removed since they were deemed to be culturally irrelevant.

Like the original SF-QTAC-PREM acute care, our new questionnaire found a 2-factor structure, however, “Information and care” formed the first latent variable, and a second latent variable regarding “safety and unfair treatment” was formed. Cross loadings were considered in the interpretation of factor loadings as overlapping constructs as new factors were identified in the new version, however, factors loadings did not indicate overlapping constructs. “Offered to discuss emotional needs” was not included in the psychometric testing by Bobrovitz et al (2017) due to major revision of the item, however, it was placed within the subscale “Information and communication”. We included the item during the Exploratory Factor Analysis, but the item showed a poor correlation with other items in both factors. Hence, it was placed as a stand-alone item in the final 2-factor structure. We agree with Bobrovitz et al [27] that it conceptually and clinically fits within the subscale but were unable to statistically demonstrate such a fit.

Compared to the original SF-QTAC-PREM acute care, the Cronbach’s alpha scores in the new Swedish version were overall higher. This might be explained by the fact that even though the new Swedish questionnaire also consists of two factors, they comprise different items in each factor compared to the original SF-QTAC-PREM acute care. The lower scores in the original were items in the Factor “Information and communication”. The Spearman’s r correlations were higher in many items in the new questionnaire compared to SF-QTAC-PREM acute care (27). All scores except one had, according to Spearman’s r correlation, a fair correlation which was positively significant, potentially due to a ceiling effect. The Spearman’s correlation for hygiene was lower, but significant. It suggests that there is a positive correlation to the global rating score, but that its relationship may be complicated in relation to the global rating score. Even if this item was scored poorly, the overall score – rating of quality of care – was high. The items “helped with hygiene”, “mobilised with care” and “help with pain” were lower. This may be due to factors, such as nurses’ high workload or the provision of highly specialised care. It maybe that the length of stay, which is not available for this analysis may also have had an effect on these item responses and results. As spearman’s rank correlation is not able to identify the effects of other complicated relationships, this may be an additional limitation.

Whilst most of the Cohen’s weighted Kappa coefficient and Intraclass Correlation Coefficient (ICC) estimates were high when assessing test-retest reliability, most were also noticeably higher than in the original [27]. Some items in the new questionnaire demonstrated low estimates; “pain well-controlled”, “Consistent information” and “Treated unfairly”, and these were below the coefficients in the original. Several factors may affect this response shift. Firstly, the response rate for retest was only 37%, and as such this self-sampling within the group may have affected the result [45]. Secondly, recall bias for “Pain well-controlled”, “All injuries explained” and “Treated unfairly” may be difficult/unable to accurately recall. Differential recall bias – where the recall is dependent on the present outcomes being experienced [46] and even recalibration of experience after previously highly challenging events [47] could affect the retest reliability results. Recalibration of experiences may affect pain ratings, however pain has also been linked to poor discharge information and lack of skills to perform effective independent pain relief at home [6]. The reliability scores using weighted Kappa and Intra Class Coefficient may also have been influenced by the participants level of education, where over half had a university level of education or higher. Higher education levels have been shown to lower reliability in longitudinal studies perhaps due to higher cognitive function and a greater ability for learning [48]. Other external factors many have influenced the results, such as the questionnaire length, understandability of the questions, practice effects and time and/ or place of completion, age (median age 58 years), gender (66% male respondents) and environment, (home environment or even during/after rehabilitating or another type of care provision) – may influence the results [46].

### Strengths of the study

A bilingual expert group was involved in the translation and cultural adaptation process to increase rigour and establish experiential and conceptual equivalence in the cultural adaptation for the survey. Reducing bias in responses was further achieved by using formats and language in well-established national patient surveys and generic PREMs in the Swedish language (construct irrelevant variance – the way in which people commonly respond to surveys). Furthermore, by using standardised processes [29,33] in translation and cultural adaptation, an effort to minimise non-equivalence bias to the original was implemented. Meaning and context were thereby considered to ascertain a realistic translation and avoid information bias and conceptual bias.

### Limitations of the study

The study took place in a single site only, a Level 1 trauma center in a major Swedish city. In addition, the recruited patients may represent a sub-group of motivated trauma patients with more (or less) severe injuries, which may affect responses and thereby also limit the external validity and the generalizability. The study sample included a larger subgroup of men, reflecting the national statistics on trauma care, but which could induce a bias and challenges during statistical analysis. There were no patients experiencing burns as with the original study, as patients who had burns were admitted elsewhere or had other comorbidities or refused to participate. Ceiling effects may have affected the Spearman’s r correlations, however these results overall remained significant. The test-retest was performed within seven days, which may have affected recall bias. Response shift bias is difficult to avoid in the test-retest results and may, in collaboration with external factors after discharge, have contributed to lower scores to the items “Pain well-controlled”, “Consistent information” and “Treated unfairly” during the test-retest analysis.

## Conclusion

The new Swedish SF-QTAC-PREM acute care exhibited above acceptable internal reliability, good overall internal consistency scale and construct validity. Implementation of this trauma-specific PREM into structured quality and safety data collection could lead to improvements in safety, trauma care process and patient involvement.
